# A legion of potential regulatory sRNAs exists beyond the typical microRNAs microcosm

**DOI:** 10.1093/nar/gkv871

**Published:** 2015-10-10

**Authors:** Ashwani Jha, Ganesh Panzade, Rajesh Pandey, Ravi Shankar

**Affiliations:** 1 Studio of Computational Biology & Bioinformatics, Biotechnology Division, CSIR-Institute of Himalayan Bioresource Technology (CSIR-IHBT), Palampur 176061, HP, India; 2 Academy of Scientific & Innovative Research, Chennai, India; 3 CSIR Ayurgenomics Unit –TRISUTRA, CSIR-Institute of Genomics & Integrative Biology (CSIR-IGIB), New Delhi, India

## Abstract

Post ENCODE, regulatory sRNAs (rsRNAs) like miRNAs have established their status as one of the core regulatory elements of cell systems. However, large number of rsRNAs are compromised due to traditional approaches to identify miRNAs, limiting the otherwise vast world of rsRNAs mainly to hair-pin loop bred typical miRNAs. The present study has analyzed for the first time a huge volume of sequencing data from 4997 individuals and 25 cancer types to report 11 234 potentially regulatory small RNAs which appear to have deep reaching impact. The rsRNA-target interactions have been studied and validated extensively using experimental data from AGO-crosslinking, DGCR8 knockdown, CLASH, proteome and expression data. A subset of such interactions was also validated independently in the present study using multiple cell lines, by qPCR. Several of the potential rsRNAs have emerged as a critical cancer biomarker controlling some important spots of cell system. The entire study has been presented into an interactive info-analysis portal handling more than 260 GB of processed data. The possible degree of cell system regulation by sRNAs appears to be much higher than previously assumed.

## INTRODUCTION

Initially, the duplex form of sRNAs capable to form complementarity with target RNAs were found regulating the genes post-transcriptionally (1,2). Based on this observation more miRNAs were identified. At and after this point a few trend setting developments occurred: exogenous synthetic molecules causing RNA interference were called siRNAs (small/synthetic interfering RNAs), while the duplexes being produced endogenously were termed ‘micro-RNAs (miRNAs)’. In the followup, with the availability of closely discovered miRNAs, some common features like hairpin-loop formation capacity and in/exact base pairing in the stem region became most prominent miRNA features to identify the miRNA precursors. Since then, these features have been defining properties of miRNAs on which several computational tools and approaches were developed (3–6). To this date, the same belief continues for miRNA ‘precursor’ identification. However, most of the genomic regions are capable to form stable hairpin loops. Bentwich et al. reported ∼11 million hairpin loops across the genome (7). In fact, most of the previously identified properties like thermodynamic stability, sequence conservation, terminal loop size and 3′ overhang today stand challenged for not being specific enough (8,9). The earlier found miRNAs and properties defined over them appear to influence the protocols and so the concept about miRNAs in general. This had resulted into an era where reporting of novel miRNAs was almost halted. Sequences which fitted the mentioned parameters defined over already existing and abundant miRNAs and satisfied the view of a typical canonical miRNA were reported in large, saturating the databases and data-sets of the algorithms intended to discover novel miRNAs. However, this trend was broken with the advent of next generation sequencing (NGS) technologies, powerful enough to detect most of the existing sRNA sequence even with low abundance, resulting into sudden surge in the reporting of novel miRNAs (9). NGS led discoveries not only impacted the number but also the concept of miRNAs. Unlike the previous definitions for siRNAs and miRNAs, the line of demarcation between these two has blurred now. Now, siRNAs are reported to form endogenously too and capable of causing the same impact as typical hairpin loop derived miRNAs (10). A wide range of endogenous regulatory small RNAs exist within the animal cell system, originating from antisense transcripts (nati-siRNA), being generated from degradation products (rasi-RNAs) or piwi RNAs (11–14). Besides this, endogenous regulatory small RNAs are now also reported from corners which were earlier blindly filtered out from genomic studies as a practice. A lot of regulatory small RNAs including miRNAs have been shown to have origin in repetitive elements (15,16). Several non-coding RNAs like snoRNAs, tRNAs, rRNAs and other non-coding RNAs have been reported to produce endogenous regulatory small RNAs capable to influence phenotypes in vertebrates (17–19). In fact, in light of NGS driven reporting of enormous number of miRNAs in miRBase, there are many miRNAs which exhibit stark difference from typical properties of miRNAs: large variations in terminal loop size, lack of 3′ overhang, mature miRNA components coming from the terminal loop region, miRNAs without duplex partners, several sRNAs being derived from a single precursor (i.e. phased miRNAs and mORs) and miRNAs with negligible loop size (Supplementary Figure S1). Some small regulatory RNAs have been reported, being derived from totally unstructured precursors (20). The diversion from general idea of s/miRNAs is not just in terms of source but even at the level of the way they are processed. Otherwise, regardless of their origin, all regulatory small RNAs exhibit an almost common route to become regulatory sRNAs: Undergo Dicer processing, followed by loading into Argonaute complex and interactions with the target. Amid this all, recent studies suggest lack of specificity by all the three major RNAse III enzymes, as Drosha/DGCR8 system, Dicer and Argonautes exhibit several non hairpin loop precursors as their substrate, suggesting clearly a wide range of sources available to provide regulatory sRNAs carrying out miRNA like function (21–24). The degree to which these sRNAs could be regulating the cell system could be much higher than expected earlier. It is more important and relevant now to look beyond the canonical products of typical hairpin-loop precursors to fathom the regulatory impacts of sRNAs.

Having this motivation, the present work was done to locate all such potential regulatory small RNAs in human cell systems. The fundamental working ideas have been : (i) recurrence of any given sRNA across large number of experimental samples provides strong evidence for non-random existence of such sRNAs. (ii) For the given samples, if the RNA-seq/transcriptome expression data is available, it is possible to estimate the anti-correlation for expression between a sRNA and its putative targets, multiple times in a re-affirmative manner. Use of proteome abundance data in measuring anti-correlation with the target gene adds further support to this. (iii) Data from interaction sequencing techniques like AGO-HITS CLIP and CLASH provide a very high confidence experimental proof of interactions between the sRNA and targets.

For this study, we considered experimental data available for cancerous and normal states for many individuals as well as cancer types. Consideration of cancer for this study has important reasons. It has huge social, health, financial and medical impacts due to being one of the deadliest health conditions. WHO has noted that in year 2012 itself 14 million new cases and 8.2 million cancer related deaths occurred (World Wide Cancer Report 2014) and the rate of cancer is expected to increase by 70% in the coming two decades. Technically, cancer is a state of uncontrolled cell proliferation which becomes malignant. A cancerous cell has capacity to grow on itself, multiply indefinitely where loss of cell cycle control is apparent, resist all signals to stop them as well as oppose cell death and apoptosis, and become capable to migrate anywhere (25). This way, s/miRNAs become natural components for cancer studies, as their initial prominence has been due to their close involvement in cell and tissue development, control of cell-cycle states and in general association with most of the cell signaling processes (26–28). Using NGS data, the information regarding the biology of cancer has suddenly spiked (29). This has resulted into enormous experimental data availability in public domains like GEO. In the meantime, The Cancer Genomics Atlas (TCGA) has been launched which is the prime repository for all cancer related genomics data. Such availability of precious experimental data spontaneously meets the prime requirements of the present study as described above in three points. We were able to get several experimental replicates for 25 cancer types, including data for sRNAs, transcriptome and interactome, sufficient enough for confident observations regarding the possible regulatory roles of existing sRNAs in human cell system and their behavior during various cancer conditions.

## MATERIALS AND METHODS

### Experimental data

Data for small RNA seq, RNA-seq and proteins expression for 25 cancer conditions were downloaded from TCGA (http://cancergenome.nih.gov/), GEO, SRA and UCSC CGHUB (https://cghub.ucsc.edu/). RNA-seq and small RNA-seq reads for five conditions were downloaded from GEO and SRA. Complete list of various conditions and sources is available in Supplementary File S1. Expression data for genes and proteins in terms of normalized RPKM/microarray normalized expression and mass spectrometry abundance data were downloaded from TCGA. RPM for sRNAs were calculated using in-house developed scripts. Argonaute CLIP-seq data was downloaded from starBase (30) and CLASH sequencing data was downloaded from NCBI SRA under the accession ID (SRP029351). Dicer and TRBP CLIP-seq reads data were taken from (SRA ID SRP038919 and SRP050041) (31). Control, DGCR8 and Drosha knockdown related sequencing data were taken from starBase and GEO (GSE55333) (30,31). All sequencing data were checked and processed for quality as well as filtered accordingly. Genomic sequences, annotations and reference RNA sequences were downloaded from ENSEMBL (http://www.ensembl.org/index.html). To identify the transcriptionally active regions in the genome, FAIRE-seq, DNase-seq and TFBS peaks were downloaded from ENCODE (The ENCODE Project Consortium). Pathways information of each gene was downloaded from Reactome, Wikipathways, BioCyc and KEGG (32–35). Correlation coefficients were calculated using in-house developed scripts in JAVA.

Three hundred fifty-eight unique sRNA reads for tRNAs, snoRNAs, snRNAs and other ncRNAs were obtained from the different studies (36–39). Scanning against all these previously annotated data was done to pull out the overlapping cases where the regulatory sRNAs were reported previously in these studies and were also detected in the current one.

### Read mapping and target identification

Small RNA reads were merged from each experimental conditions and unique reads were selected. These unique reads were mapped to human genome build 19 (hg19) assembly using BOWTIE with maximum of two mismatches. Mapped reads were also matched to known mature miRNAs downloaded from miRBase version 21 to filter out reads mapping on known miRNAs. To filter out small RNA reads as some random product, two different criteria were applied: (i) only those reads were considered which appeared more than five times in any given experiment, and (ii) at least for two different experimental conditions. All such reads were subjected to target identification. Targets of novel regulatory small RNAs were identified using TAREF (40) and TargetScan (41). Small regulatory RNAs which were found to target genes were searched against the complete genome annotation downloaded from ENSEMBL. For this purpose, the co-ordinates of small regulatory RNAs were matched against the annotations provided in ENSEMBL, using openMPI library based in-house scripts on LINUX cluster system.

### Validation of identified sRNA:target interactions

For the validation of sRNA:targets interactions identified above, targets and partner rsRNAs were searched across Argonaute CLIP-seq data and CLASH sequencing data. Also, Pearson correlation coefficient (PCC) between a target gene's protein level, transcript abundance and small regulatory RNA expression was calculated to further verify functional relevance of these sRNAs. Argonaute CLIP-seq data was available for four Argonaute proteins namely AGO1, AGO2, AGO3 and AGO4. The reported sRNA:Target interactions were scanned through all these Argonautes’ cross linking data. Using the RNA-seq based RPKM values of target genes and RPM values for sRNAs, expression correlation was calculated between the sRNA and its targets. Since protein abundance was normalized and reported in TCGA, the correlation coefficients were calculated using normalized protein abundance and RPM values.

### Functional and pathways characterization of target genes

For each target gene, KEGG pathway information was extracted from bioDBnet (http://biodbnet.abcc.ncifcrf.gov/), and Gene Ontology (GO) data was extracted from ENSEMBL. Gene Enrichment Analysis (GEA) of target genes was done using hypergeometric test with Bonferroni correction in R. Enrichr (42) was used for functional and pathways enrichment analysis using KEGG, Biocarta and Wikipathways.

### Location and biogenesis of novel regulatory small RNAs

Novel small regulatory RNAs were mapped to human genome hg19 build using Bowtie with ‘-a’ option to report all matching loci. The annotation of loci was done using the annotation file downloaded from ENSEMBL. These loci were searched in UCSC ENCODE data for identification of transcriptionally active regions. For this purpose DNA-seq, FAIRE sequencing and TFBS data were downloaded from UCSC ENCODE database. Bedtools was used to report the overlapping loci with transcriptionally active regions. To identify the rsRNAs which were processed by DGCR8 complex, DGCR8 down-regulated sRNA reads were compared with DGCR8 expressed and DGCR8 CLIP-seq reads to identify sRNA reads processed by DGCR8. To identify rsRNAs processed by DROSHA, knockdown data of DROSHA was considered (GSM1550168 and GSM1550168). Similar steps were taken to identify the Dicer (GSE55324) and TRBP (GSM1548746 and GSM1548747) dependent rsRNAs which were scanned in their corresponding CLIP-seq read data. The genomic sequences with 100 bp left and right flanking regions from the start point of the given sRNAs were analyzed for hairpin-loop and miRNA precursors structure support using miREval (43) and RNAfold (44).

**Figure 1.**
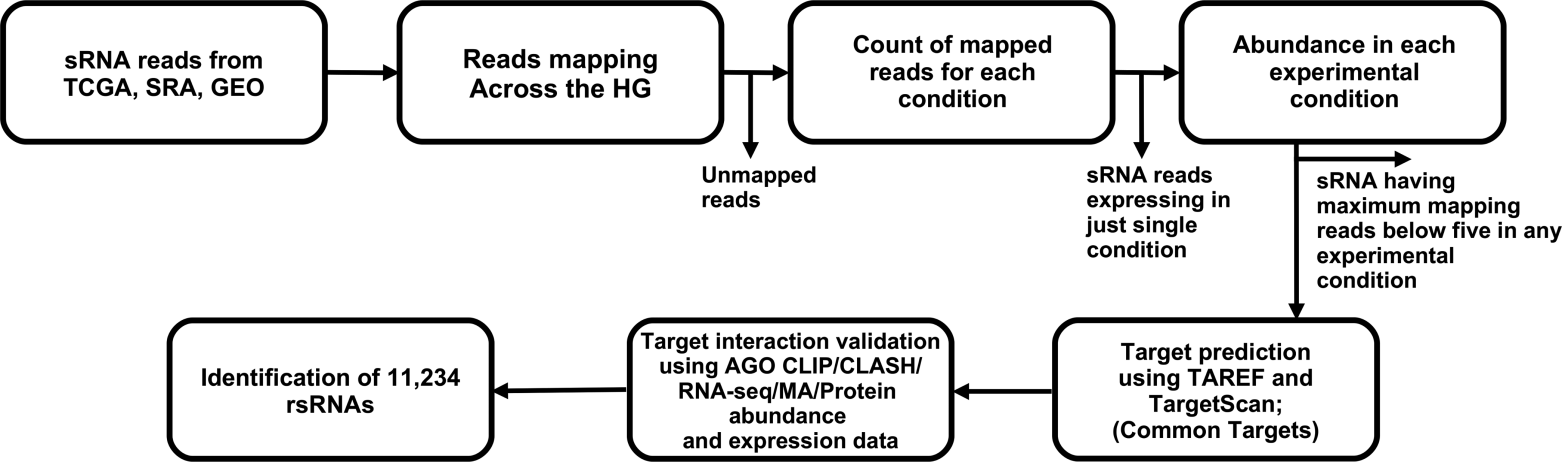
The basic work-flow adopted to identify the regulatory small RNAs. Following the same fundamental steps the potential rsRNA (11 234) were reported. These regulatory sRNAs exhibited potential target interaction of high confidence and were found consistently associated with Dicer, TRBP and AGO line of sRNA processing and target loading path, a signature of regulatory sRNAs.

Figure 1 Summarizes the basic work-flow of the present study. All statistical tests were performed using open source software for statistical analysis, ‘R’ (R Core Team).

### Experimental validation of regulatory sRNAs

#### RNA purification

Total RNA was isolated from cultured cells of all the four cell lines, A549 (Lung Adenocarcinoma), MDA-MB-231 (Breast cancer), HeLa (Cervical cancer) and MCF7 (breast cancer), using Trizol reagent (Invitrogen, Life Technologies Corporation, Carlsbad, CA, USA), according to the manufacturer's instructions. Subsequent purification using an RNeasy Kit (Qiagen, Hilden, Germany) was carried out to remove residual aromatic compounds. One μg of total RNA was used to make cDNA separately for rsRNAs and gene specific qPCR.

#### cDNA synthesis and quantitative real-time PCR (qPCR)

*rsRNA*: cDNA synthesis was done using QuantiMir RT Kit (Small RNA Quantitation system) from System Biosciences (SBI) following manufacturer's protocol. Briefly, small RNAs present in the total RNA were tagged by a Poly-A Tail, followed by annealing of anchor oligo-dT adaptor to the poly-A tail. These were then carried forward for cDNA synthesis resulting in pool of anchor-tailed small RNAs. cDNAs were checked by end-point PCR with kit supplied universal reverse primer and miRNA-specific forward primer (human U6 used as control). The cDNAs were diluted 1:20 before being used for qPCR.

*Gene*: Total RNA was converted to cDNA using random primers and High Capacity cDNA Reverse Transcription (Applied Biosystems, Life Technologies Corporation, Carlsbad, CA, USA) as per manufacturer's instructions. The resultant cDNA was diluted 1:10 for qPCR, subsequent to check with a control β-actin primer.

qPCR was performed using KAPA SYBR^®^ FAST qPCR Kit (KAPA Biosystems) on a Roche LightCycler^®^ 480 instrument (384-well), using default parameters. HPLC purified primers were synthesized from Sigma-Aldrich for this work. Primers used for qPCR of rsRNA and gene are listed in Supplementary File S2.

## RESULTS AND DISCUSSION

### Identification of putative regulatory sRNAs and their interactions using multilayer validation

**Figure 2.**
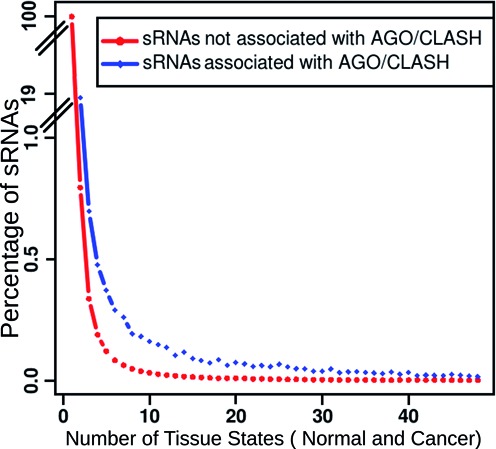
Distribution of small RNA reads across the 25 cancerous and respective normal conditions with percentage of sRNA:target interactions identified in AGO-HITS CLIP/CLASH data. As evident from the plot, the fraction of sRNAs found associated for interactions were higher for multiple experimental conditions when compared with the fraction of the population of unassociated sRNAs. Also, in general, there was a sharp decline in the number of total sRNA when occurrence in more than single experimental condition was considered, suggesting suitability of recurrence as an effective initial filtering parameter to identify the rsRNAs.

**Figure 3.**
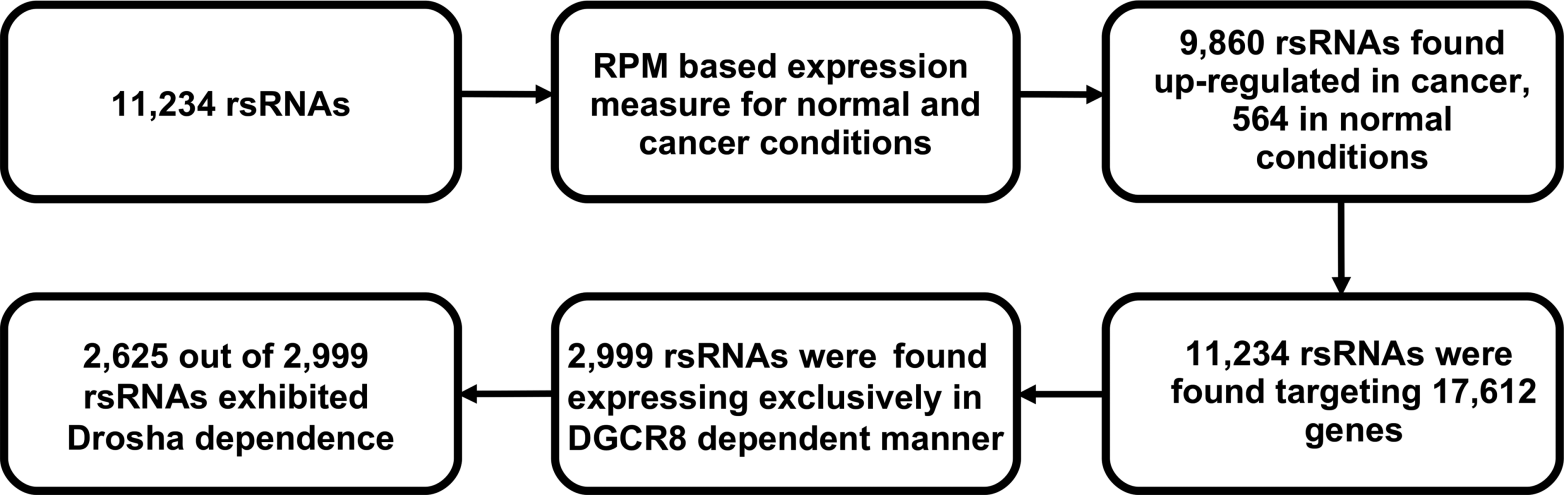
Work-flow illustration of analysis performed on regulatory small RNAs with respect to DGCR8 dependent processing, a step involved in canonical miRNA processing. A total of 2999 rsRNA appeared as potential miRNAs for which strong DGCR8 and Drosha association supports were available along with traditional structural support. 2501 of these potential miRNA candidates exhibited all the three Drosha processing associated motifs.

Small RNA reads from all considered cancerous and normal tissue-specific conditions were pooled to identify the unique reads. These unique reads were mapped to human genome assemblies (hg19, hg18). A total of 523 540 761 unique reads were obtained from sRNA-seq read data, of which 317 890 669 (60%) reads mapped across the human genome with 100% match. The number of reads were reduced to 23 369 669 (7%) when only those reads were considered which were expressed in two or more conditions. 10 789 006 (45%) unique reads out of 23 369 669 unique reads were such reads which exhibited abundance of at least five reads. These 10 789 006 small RNA reads were considered as the final set of small RNAs for further analysis. These sRNA were used to identify targets using TAREF (40) and TargetScan (41). A total of 8 276 923 (76%) out of 10 789 006 small RNAs were found to target 17 612 genes with maximum of two mismatches in interaction patterns between target and small RNAs. An analysis of these sRNAs against their presence across various experimental conditions and AGO cross linking/CLASH data suggested that the number of sRNAs falls steeply from their presence in single experimental state to presence in at least two experimental conditions (Figure 2). As can be observed from the plot, for all the recurrent experimental conditions, the fraction of sRNAs found associated with AGO/CLASH was higher than the fraction observed for unassociated sRNAs. This suggests that the sRNAs found associated with AGO/CLASH data had larger fraction of its population in multiple experimental conditions than those which were not found associated with any interaction data. This all provided the reasoning to consider only those sRNAs which existed in at least two experimental conditions and were associated with AGO/CLASH data. In overall, 183 601 unique sRNAs were found associated with AGO/CLASH, while 10 224 907 unique sRNAs were found not associated with AGO/CLASH and were recurring at least in two experimental conditions. A list of sRNAs not associated with AGO/CLASH interaction data but exhibiting recurrence has been provided at the complementary ‘mythology’ portal associated with this work. Though the sRNAs which exhibited recurrence but were not found associated with any interaction evidence may exhibit targeting capacity (8 093 322 of such sRNAs returned common targets for TargetScan and TAREF runs) in some other experimental conditions, in the present study for further analysis only those sRNAs were considered which exhibited recurrence as well as association with AGO/CLASH interaction data. From here on only those sRNAs were considered which exhibited two fold or above differential expression at least in any single state. This way, a total of 11 234 potential novel regulatory sRNAs were identified. 9860 regulatory small RNAs displayed higher abundance in cancerous conditions, whereas 564 rsRNAs showed higher abundance in normal conditions, remaining 810 rsRNAs were equally over-expressed in normal and cancerous conditions. The identified potential regulatory sRNAs were scanned for their presence across the Dicer CLIP-seq data (GSE55324) to fathom the number of such sRNAs being processed by Dicer. A total of 9316 potential rsRNAs were found associated with Dicer in its CLIP-seq data (31). When analyzed against the Dicer knockdown data (GSM1550166, GSM1550167) comparing the wild type condition, 8400 potential rsRNA were found being formed exclusively in the presence of Dicer. Having support from two entirely different methods for Dicer led generation of sRNAs, these sRNAs were further analyzed for their association with TRBP (GSM1548746 and GSM1548747). TRBP is an important mediator in RNA silencing process, where it mediates the transfer of regulatory sRNAs from Dicer to Argonaute-RISC complex to cause RNA silencing. TRBP CLIP-seq data was scanned for the presence of the potential rsRNAs and it was found that 9100 potential rsRNAs were associated with TRBP. All these rsRNA candidates could be clustered into 12 different length based clusters ranging from 17 bases long to 28 bases long. The highest number of sRNAs were found having length of 17 bases (4,312) followed by 18 bases (2,174) and 21 bases (1,644). Average read copies of the rsRNAs was found to be 37.53, spread in the range of 6 to 1457 observed maximum read copies in any given experimental condition. All these observations also suggest that it is highly improbable that the reported rsRNA candidates could be a random degradation product. These novel 11 234 potential rsRNAs were also searched against the DGCR8 knockdown and over-expressed HITS-CLIP data to identify the possible DGCR8-DROSHA mediated rsRNAs. Those candidates were considered as processed by DGCR8 if found exclusively present in DGCR8 expressed and cross linked data only and totally absent in DGCR8 knockdown condition. 2999 (26.69%) of the potential rsRNAs were found exclusively expressed in the presence of DGCR8. Similar analysis was performed for Drosha dependent existence of these sRNAs. It was found that 2625 out of 2999 such sRNAs (87.58%) were formed exclusively in the presence of Drosha and were totally absent in Drosha knockdown condition. Therefore, all these rsRNA candidates displayed distinct dependence upon Drosha-DGCR8 system for formation, which is a trademark of canonical miRNAs. All such sRNAs were mapped to the genome and genomic sequences starting from 100 bp 5′ and 3′ flanking regions from its start position were extracted to find out if such sRNAs existed within the stem of any stem-loop precursor structure. For 2204 sRNAs miRNA precursors were found supported by miREval (43) (data available at associated portal). 1125 such sRNAs were present almost perfectly within the stem region. Figure 3 summarizes the findings made in this section. Recently, Auyeung et al. (31) explored for novel features of Drosha processed miRNAs and they found predominance of certain motifs in pri-miRNA sequence and structure context around the Drosha processing sites. It included the predominance of CNNC motif around 17th nucleotide downstream of the precursor's 3′ end, U at –14th and G at –13th position from the 5′ end of the precursor in independent manner, where UG enrichment was observed the most, and the presence of UGU/GUG/UGUG motif within the precursor region, mostly in the apical terminal loop. The identified 2999 potential miRNAs' extended sequences covering potential pri-miRNA regions, as mentioned above, were scanned for all these positional motifs. A total of 2501 miRNA candidates reported the presence of all these three major signatures together, further supporting their genuine candidature for being miRNA. Supplementary File S3 provides information about all these 2,999 potential miRNA candidates. Additional information has also been provided at the companion site.

As mentioned above, 17 612 different targets (unique genes) were predicted commonly by TargetScan and TAREF systems. In order to validate these target:small RNA interactions, the interactions were first searched against the Argonaute sequencing data downloaded from starBase (version 2) (30). A total of 149 344, 150 049, 145 561 and 148 251 novel putative small regulatory RNA:target interactions for 8036, 6247, 8342 and 7196 unique rsRNAs and 14 265, 12 448, 14 438 and 13 674 unique targeted genes were validated using AGO1, AGO2, AGO3 and, AGO4-CLIP sequencing data (Supplementary File S4), respectively (Supplementary Figure S2). This also suggested that majority of the potential regulatory small RNAs could be loaded into the different AGO complexes and target number of genes. From the analysis, it was found that many of these novel putative regulatory small RNAs were simultaneously loaded into different Argonautes instead of showing any specific preferences, exhibiting concordance with earlier finding that sRNAs don't display any major sorting preferences for the various Argonautes (42–46). Apart from CLIP-seq data, CLASH-seq data was also used for the validation of rsRNA: target interactions. CLASH-seq data gives more precise information about the interactions as the interactions are arrested through ligation of the target and targeting small RNA. Using CLASH-seq data, 16 371 unique genes were found being targeted by 10 048 putative rsRNAs, comprising 474 770 unique target interactions (Supplementary Figure S2). In overall, all these steps in analysis give a complete flow illustration with strong serial evidences, starting from processing by Dicer, subsequent transfer and loading by TRBP and final acceptance of the processed rsRNAs by Argonaute.

To add another layer of validation for their existence and their possible regulatory presence, correlation between the expression patterns for the identified interacting rsRNAs and their target partners was assessed using RNA-seq as well as protein abundance data. Protein abundance data was available for only 192 genes in TCGA for different cancerous conditions whereas gene expression data from microarrays and RNA-seq was available for 17 488 and 17 814 unique genes, respectively. Since sRNAs suppress the expression of the target gene, an anti-correlation was expected between the targeting rsRNA and target. For some cancer conditions either the expression data was not available or the data was restricted by TCGA. Protein and miRNA expression anti-correlation estimation was performed for 4776 patients for 15 cancer conditions for which data was available. Targets validated using protein:sRNA co-expression identified many important small regulatory RNAs which target genes involved in important functions such as tumor enhancer or tumor suppressor genes (Supplementary File S4). Out of 192 genes having protein abundance data, significant anti-correlation for expression was observed with 131 unique genes as the targets for 9446 rsRNAs.

Another level of validation was obtained by performing sRNA and target genes expression analysis using their read counts. A total of 3013 cancerous and normal tissue based experimental conditions were considered. For some cancer tissues the RPKM based gene expression data was not available. In such situation the microarray expression data from TCGA (for 137 cancer and normal conditions) was used. Details of s/RNA-seq and microarray based expression study are given in Supplementary File S5. Significant (*P*-value < 0.05) and high inverse correlation (PCC > |–0.5-> –1|) coefficient between target genes and targeting rsRNAs was observed for 11 234 sRNAs and 17 093 unique genes for sequencing data, while the microarray data showed 8931 sRNAs having strong anti-correlation with 16 020 target genes. In order to assess if targeting could be associated with anti-sense phenomenon, from the list of targeting rsRNAs those rsRNAs were identified whose biogenesis locus was coinciding with the binding site in the target gene and were originating from the opposite strand of the target. From this analysis, a total of 1185 unique rsRNAs were identified regulating 211 unique genes (Supplementary Figure S3). Also, it was found that 10 968 rsRNAs targeted UTRs of 13 264 genes with a total of 932 266 interactions.

**Figure 4.**
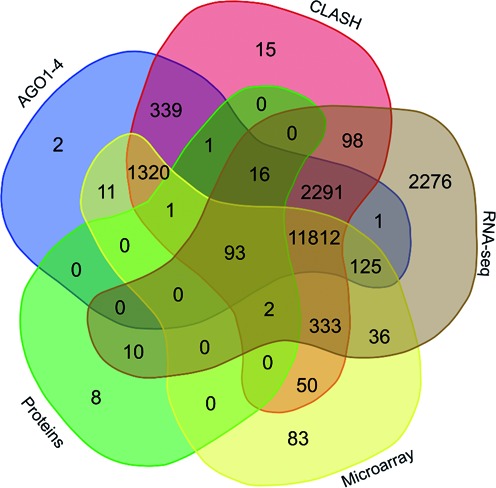
Overlapping interaction supports from different platforms for the identified rsRNAs. The figure shows Venn diagram representing target genes identified using AGO1–4, HITS-CLIP data, CLASH-sequencing, RNA-seq based digital gene expression profiling, microarray based gene expression profiling and protein expression based target identification. Most of these interactions reported through different platforms concurred with each other, giving strong evidence for interactions of rsRNAs with their potential targets.

Figure 4 illustrates the level of agreement between the different validation approaches for the identified rsRNA and target interactions as well as the location of target genes identified exclusively using AGO CLIP-seq, CLASH-seq and common in both techniques. As apparent from this study, for most of the interactions two or more methods agreed, strongly suggesting the regulatory existence of these sRNAs. These novel sRNAs showed significant negative correlation with the target genes identified using digital gene expression data and protein expression data. Many of the target genes were found important for pathways related to cancer, with names like BRCA2, p53, Rb, Myc, 14–3–3 epsilon, CycD and CycE.

**Figure 5.**
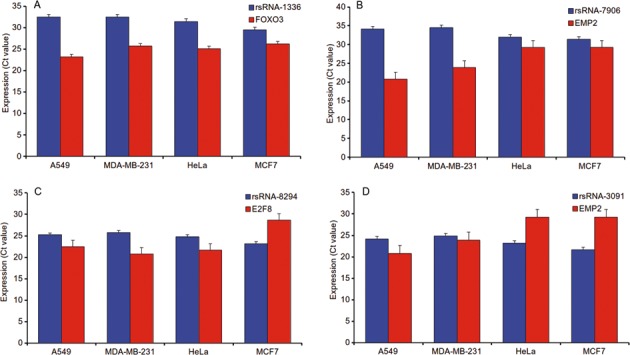
qPCR based validation of rsRNAs and target genes. Functional validation of 22 rsRNAs and their corresponding target genes was done using qPCR across four different cell lines (HeLa, MDA-MB-231, A549, MCF7). The targets exhibited significant coefficient of anti-correlation with their targeting rsRNA. Figure 1(**A**) plots the expression levels for rsRNA 1336 and its target gene FOXO3, (**B**) plots the expression levels for rsRNA 7906 and its target EMP2, (**C**) plots the expression levels for rsRNA 8294 and E2F8, and (**D**) plots the expression levels of rsRNA 3091 and EMP2.

Finally, 22 potential rsRNAs identified through analysis above, were selected for experimental validation for their expression and potential target regulation, following the procedure described in the methods section. The experiment was done in triplicates in four different cell lines, namely, A549 (Lung Adenocarcinoma), MDA-MB-231 (Breast cancer), HeLa (Cervical cancer) and MCF7 (Breast cancer). qPCR confirmed differential expression levels for 19 out of 22 rsRNA and 14 out of corresponding 22 target genes across the cell lines. Rest did not work experimentally under the condition tested. One exception is *GK5*, where rsRNA2474 targeting the gene did not work in any of the cell lines. Significantly, it was observed that 8 out of 13 target-rsRNA pairs (∼62%) displayed strong inverse correlation. For the remaining pairs for which strong anti-correlation could not be found, it might be plausible that they show such regulation in different tissue origin cell lines, tissue *per se* or there could be multiple targeting sRNAs involved. For some cases, the target genes were expressing in less than 3 cell lines, making it insufficient for correlation measure. Importantly, we found that most of the rsRNA : target gene pairs exhibited inversely correlated Ct values in the same background of the tissue in which they were discovered. Few prominent examples being rsRNAs 7906, 9345, 4150, 3790, 3091, 2734 and 1336. All these rsRNA were discovered in the lung tissue and they showed lower expression in A549 whereas the target genes were expressed in higher quantity. Same goes for rsRNA 8294 and its target gene expression in breast cancer cell line MCF7. We also observed that rsRNA 3091 which showed expression in breast cancer in addition to lung, was found to reflect the same pattern of anti-correlated expression in MCF7, but not in MDA-MB-231. This highlights the possible role of additional factors leading to rsRNA-target regulation. The importance of tissue background of expression is also highlighted by non-directional levels of rsRNAs 9338 and 551 with their target genes. Overall, the experimental results elucidate the existence and differential expression patterns of the queried rsRNAs and their corresponding targets, suggesting their possible regulatory roles in tissue-specific manner (Figure 5; Table 1). The associated information has been made available in Supplementary File S2.

**Table 1:** Experimental validation of rsRNAs and associated targets using qPCR

rsRNA/Gene	A549 (Lung adenocarcinoma)	MDA-MB-231 (Breast cancer)	HeLa (Cervical cancer)	MCF7 (Breast cancer)	PCC
rsRNA-1336	32.48	32.46	31.41	29.43	-0.62
*FOXO3*	23.17	25.73	25.07	26.21	
rsRNA-3091	24.17	24.81	23.19	21.66	-0.77
*EMP2*	20.80	23.90	29.22	29.26	
rsRNA-3790	26.82	27.60	26.55	25.02	-0.56
*TAF8*	19.94	22.89	23.08	24.71	
rsRNA-5402	31.20	32.02	29.52	28.50	-0.96
*ATM*	24.48	22.24	26.22	NA	
rsRNA-7906	34.15	34.53	31.96	31.4	-0.91
*EMP2*	20.80	23.90	29.22	29.26	
rsRNA-8294	25.20	25.77	24.79	23.11	-0.96
*E2F8*	22.42	20.73	21.61	28.62	
rsRNA-9338	32.28	33.55	31.82	29.70	-0.98
*TERF1*	26.46	25.66	26.31	27.32	
rsRNA-9345	35	35	34.03	32.16	-0.78
*BRCA2*	22.76	26.78	33.01	33.18	
rsRNA-3091	24.17	24.81	23.19	21.66	-0.66
*TAF8*	19.94	22.89	23.08	24.71	

The table summarizes the expression level differences for rsRNA and target genes along with their Pearson Correlation Coefficient (PCC) for expression values across different cell lines. Out of 22 rsRNAs selected for experimental validation, 19 rsRNAs were found reasonably expressing in the given cell lines.

### Repeats, introns and ncRNAs have major stakes in regulatory small RNAs

It is an irony that most of the miRNA discovery methods plainly discard the reads displaying any repetitiveness and association with repeats. This happens despite of continuous reports suggesting that repeats are not junk but genomic goldmine having critical roles. This study reports an exclusive association between the regulatory sRNAs and repetitive elements. It was found that these small regulatory RNAs were originating from multiple loci mainly belonging to repetitive elements, intronic region and ncRNA regions (Table 2; Supplementary Figure S4; Supplementary File 6). The abundance of intronic source for these small regulatory RNAs concurs with the fact that a large amount of already reported regulatory small RNAs like miRNAs originate from the intronic regions (47–49). The current version of miRBase (version 21) has ∼46% miRNAs from the intronic regions (50). However, what looked more fascinating was the high share of repetitive and noncoding elements. Many diverse forms of endogenous siRNAs and non-canonical miRNAs have been reported recently (51–54). The highest numbers of regulatory sRNAs were found belonging to Alu elements, ERVs, LINEs and hAT elements. A number of previous works have already pointed this out repeatedly that numerous such regulatory small RNAs could have origins in retro and transposing elements of the genome (16,55,56). For all the novel identified rsRNAs in this study their corresponding coordinates were identified and mapped to the transcriptionally active coordinates identified by ENCODE. Overlaps between transcriptionally active regions and rsRNAs were identified using ‘intersect’ module of BED tools. Out of 11 234 rsRNAs, 10 698 (95.22%) rsRNAs were identified whose coordinates did not overlap with any coding region. For these 10 698 rsRNAs, the coordinates of 7802 (72.92%) rsRNAs were found overlapping with transcriptionally active regions. The majority of such rsRNAs were found coming from ncRNAs and Alu subfamilies (Supplementary File S6). Most of ncRNA associated rsRNAs were coming form LINC RNA (620) (Supplementary File S6). Among these all, the case of Alu elements becomes most notable as 4592 different sRNAs were found originating from the Alu elements. More interesting was to observe the distribution profile of these sRNAs across the length of Alu consensus, which followed almost a conserved non-random pattern of sRNAs abundance for a number of different experimental conditions (Supplementary Figure S5A). The sRNAs originating from Alu displayed absolute conservation of profiles across the individuals, which was also seen distinguishing significantly between the normal and cancer samples for many cancer types. A series of *t-tests* between cancer v/s normal conditions gave consistently significant *P*-values (*P* < 0.05) for this observation, suggesting the sRNA profile originating from Alu differ significantly between cancer and normal states. Previous studies as well as structural analysis of Alu suggest that they are potent to generate siRNAs through Dicer like endonuclease activities. The association of Alu derived sRNAs with AGO proteins and CLASH data found in this study supports the regulatory roles of these sRNAs and reasons for the observed non-random pattern of Alu derived sRNAs. In the absence of high number of individual samples, such sRNAs would be otherwise rejected as a random product or sequencing artifact. However, with consistent evidence of similar and distinctive patterns across many individual sequencing data for normal and cancer states it is tough to dismiss such sRNAs as random products. With the availability of high throughput sequencing data, some recent studies have already reported the display of similar kind of non-random pattern of sRNA biogenesis from other non-coding RNAs exhibiting regulatory roles in cancer conditions (57). Percentage of reads distribution on rsRNAs coming from repeats were also calculated. This analysis was performed to normalize the rsRNA reads as a given rsRNA coming from repeats could map on multiple loci. It was found that rsRNAs from ERVL and Alu families were distributed non-randomly across the genome followed by other repeat families (Supplementary Figure S5B).

**Table 2:** Genomic annotation of rsRNAs loci identified in the study

Genome annotation	Number of rsRNAs	Percentage of rsRNAs
Repetitive elements	5,078	45.20
ncRNAs	680	6.05
Intergenic regions	73	0.64
Introns	5324	47.39
Exons	99	0.88

Introns and repetitive elements emerge as the major sources for rsRNAs. Though repeats and multiple mapping reads are simply excluded from the usual miRNA discovery protocols, the findings here suggest repeats having a big stake in regulatory sRNAs.

The identified potential rsRNAs were also scanned against some previously reported cases of regulatory sRNAs from different non-coding elements like tRNAs, snoRNAs and other ncRNAs. This study was an apt opportunity to provide strong evidence and support to such previously identified regulatory small RNAs. A recent work by Cole et al. (38) compiled several such previously reported regulatory sRNAs and performed high throughput data backed computational analysis to confirm as well as report such regulatory sRNAs. The same set of regulatory sRNA data was scanned to find if support for them existed in the present study. They had reported a total of 358 regulatory sRNAs, out of which 125 sRNAs were found overlapping with the rsRNAs reported in the current study. Remaining 233 sRNAs were found mapping to the sRNAs which had not qualified on the series of filtering criteria mentioned above which included clauses like association with AGO, presence in CLASH data, abundance of at least five reads and differential expression in at least one experimental condition. Most of these sRNA were found absent in any interaction sequencing data. It was also found that several of these reported sRNAs were extremely poor in expression or had no differential expression. A complete breakup has been provided in Supplementary File S7.

### The potential regulatory sRNAs distinguish between cancer and normal states

One of the most interesting findings made in this study has been the identification of certain regulatory small RNAs as the markers of the cancer states studied here. The history of small regulatory RNAs itself started with discovery of their roles in cell and tissue development whose reporting increased only with time. A number of s/miRNAs have been implicated in cell differentiation processes (26–28), tissue-specific growth and ominous control of most critical regulatory networks of cell system (53). Any change in the basic fundamental character of any of these systems may lead to serious intervention in normal development and growth leading to unwanted outcomes like cancer. Thus, a lot of miRNAs have been found strongly associated with cancer states.

In the present study, several potential regulatory small RNAs were found significantly differentially expressed between normal and cancer states. The differential expression was evaluated across large number of individual samples, followed by *t*-test for significance between normal and cancer state samples for every cancer condition studied here. Supplementary File S8 provides the list of top 10 over-expressed small regulatory RNAs for the compared conditions for cancer and respective normal states along with the functional enrichment classes for each cancer type. The full details including the list for all over-expressed sRNAs can be found on the ‘Mythology of ‘micro’-RNA’ portal described below. All these over-expressed miRNAs displayed a strong negative expression correlation value (<–0.5) with their corresponding targets. The functional enrichment analysis over their targets provided us the insight that most of the targets of these regulatory small RNAs were associated with cell development, its life cycle, growth and apoptosis (Supplementary File S8).

Though this analysis has provided a huge amount of information, it would be difficult to discuss all of them here. The readers are suggested to explore the ‘Mythology of ‘micro’-RNAs’ portal for details. However, as an instance, it would be interesting to discuss the case of regulatory sRNA 9881. This small regulatory RNA was found over expressed in almost all cancer states studied here, suggesting about some central points being affected by this small RNA. There were 20 different target genes which were found strongly negatively correlated to its expression (Table 3). A closer analysis revealed that the target genes were enriched for pathways critical for cell development and cancer at the interfaces of diverse pathways (apoptosis, cell death, p53 signaling, hiv-1 nef, caspase cascade, TLR, TNFR-1 signaling and FAS pathway), reasoning why the regulatory sRNA 9881 was found abundant in most of the studied cancer conditions. Supplementary File 9 presents details of all the target genes of rsRNA-9881. A motion chart link (http://14.139.59.221/∼scbb/support_site/all_data/rsRNA-9881-n.html) describes the regulatory implications of regulatory small RNA 9881 and expression relationships between rsRNA-9881 and its targets. An interaction network was built with these 20 target genes to understand their relative standing in the networks. Certain genes emerged as central to several critical processes. The most connected genes were *CASP8, OLA1* and *SUV39H2*. In overall, all these 20 genes were found closely associated. *CASP8* is critical for *MAPK* signaling cascade, apoptosis modulation, insulin signaling and tight junction formation. *DDX52* associated genes were found enriched for pathways in cancer, *MAPK* Signaling and apoptosis. *FKBP* associated genes were found enriched for genes involved in multiple cancer pathways, insulin signaling, B-cell receptor signaling, focal adhesion, *WNT* signaling pathways. Adipocytokine signaling, insulin signaling, cancer related pathways, EBVL MP1 signaling, TOR signaling were found enriched in *MAP3K* connected genes. The *MBD4* associated genes had broad range of impacts, affecting FAS pathways, apoptosis, cell cycle, notch signaling, pathways in cancer, glycine-serine-thronine metabolism, cysteine-methionine and other amino acids metabolism. *MBD4* has already been reported as a gene which is omnipresent as a tumor suppressor gene whose down regulation is related to several types of cancer. The genes associated with *MECR* were found enriched for adipogenesis, adipocytokine signaling, fatty acid biosynthesis, *PPAR* signaling and pathways in cancer. *MECR* associated genes have been found affected in cancer while *MECR* is reported as the last step gene of fatty acid biosynthesis pathway, seated at mitochondria, but also standing as the final connecter to the nuclear signal through *PPAR* (58). Another target gene, *MRTO4*, is associated with ribosome formation and translation rate, which was reported to be involved with *PKR* to control the cell cycle process in normal conditions (53). The genes interacting with *MRTO4* were found enriched for chemokine signaling, cytoplasmic ribosomal proteins, inflammatory response, adipogenesis, focal adhesion and senescence and autophagy. Another target gene, *OLA-1*, is a newly found ATPase member of YchF subfamily GTPases whose functions are still not clear. It has been found critical in centrosome and spindle pole formation (59) and in cell survival during stress (60). The genes interacting with *OLA-1* were found enriched for oxidative phosphorylation, ETC, adipogenesis, gluconeogenesis, focal adhesion, glutathion metabolism and fatty acid metabolism. For the target gene RNF213, the genes associated with functions like focal adhesion, pathways in cancer and endocytosis were most prominent. However, there is not much information for *RNF 213* and its possible roles. Another target of rsRNA 9881, *SUV39H2*, is a histone methyltransferase gene, important for transcriptional silencing of genes through H3K9 methylation and transcriptional silencing during meiotic prophase, was found associated with cancer states (61). The interacting genes with *SUV39H2* were found prominent for regulation of actin cytoskeleton, purine metabolism and focal adhesion. Target gene *TACC1* down regulation and aberrant splice variant cases have been reported associated with cancer (62). The genes interacting with *TACC1* were found most prominent for axon guidance and meiosis. Target gene *YEATS2* has been reported to be associated with bone tumors (63), though not much is known about this gene. The interacting genes for *YEATS2* were found prominent for purine metabolism, RNA transport, notch signaling, insulin signaling, *RIG-I* like signaling, osteoclast differentiation etc. On collective consideration of all the interacting genes for the found targets for rsRNA 9881, the most common process found across all these genes was adipogenesis, suggesting strongly that systems associated with fatty acid metabolism and fat cell formation appear as the central of most of the cancer states. Findings here get support from the recent studies which have demanded strong attention for identification of fatty acid metabolism pathways as one of the most affected processes during cancer (64,65). One final observation has been that the expression patterns of several of these regulatory sRNAs and their relationship to cancer states were relative to the tissue type. This could be contextual to the abundance of other regulatory small RNAs. A proper detailed analysis is required to find out these relationships between the small regulatory RNAs and onset of cancer states. The findings made here open a scope to decipher the cancer states where these potentially regulatory small RNAs would be very helpful as markers.

**Table 3:** The most significant targets of rsRNA 9881 and their associated anti-correlation coefficient and significance values

Target gene id	Anti-correlation coefficient	p-value
*SYT11*	0.995	1.49E-129
*MBD4*	0.971	7.53E-080
*MRTO4*	0.958	1.09E-069
*PGPEP1*	0.939	1.12E-059
*YEATS2*	0.906	2.17E-048
*CYP20A1*	0.901	2.96E-047
*DDX52*	0.893	4.76E-045
*FKBP14*	0.872	1.17E-040
*RNF213*	0.871	1.91E-040
*SUV39H2*	0.860	3.04E-038
*HSH2D*	0.809	1.44E-030
*CASP8*	0.808	1.53E-030
*MAP3K15*	0.807	2.04E-030
*MECR*	0.761	2.86E-025
*FBXO27*	0.752	2.33E-024
*ALDH8A1*	0.746	8.55E-024
*TACC1*	0.723	8.66E-022
*CLDN18*	0.681	8.66E-022
*OLA1*	0.634	1.22E-018
*CEACAM5*	0.621	1.24E-015

All these targets exhibited strong inverse expression correlation with rsRNA 9881. Most of these targets were found associated with pathways and processes critical for cell development and cancer. Interestingly, most of them converge to adipogenesis and focal adhesion, which are considered as important factors in cancer development. rsRNA 9881 is among those rsRNAs which were found abundant in most of the cancer conditions studied here.

### The potential regulatory small RNA information portal

One of the important points of this study has been the amount of high throughput data considered to identify regulatory small RNAs and their impacts. While doing so, various experimental data from different platforms were pooled together, analyzed and were related to each other in a meaningful manner. Handling such data and pull out meaningful interpretations require a proper structuring and representation of the data and information tools. In this regard, a number of visually rich and useful representations have been made available as a complementary material at the associated portal (http://14.139.59.221/mythology/index.php) where a user could delve into the details. The portal has been built using next generation web development package JS, D3, Python, PERL, JSON, HighCharts and MySQL, installed over a Linux server. A user can browse the portal through several entry points: by searching for terms, browsing for rsRNAs or by directly opting for the various result sections which in turn transfer the user to entire tool sets, look into the pathways details, and select various cancer states. A wall page contains clustering of the rsRNAs on the basis of seed similarities, which provides dynamic image system and enrichment analysis for the targets of the selected clusters and sRNAs. The provisions of dynamic expression analysis is given where any number of experimental conditions could be selected along with sRNA of choice and its targets. Search can be performed using single term as well as in batch mode. Search results may be explored with two options: Annotation Viewer and Expression Search. On selecting Annotation Viewer, an annotation chart with visual genomic representation of the selected query is displayed. From here, all information for a target site and rsRNA can be accessed. Whereas, selecting expression chart button redirects the user to multiple selection input option page. From the page user can select multiple filters like support type, cancer type, sample type, number of individual and user choice plot selection (Supplementary Figure S6, http://14.139.59.221/mythology/supplementary/). The rsRNA browser section facilitates several searches and analysis at single place, where a user can look for a particular rsRNA, its experimental evidences, perform on the spot target and associated expression analysis, their enrichment for GO and pathways, and perform up to three step network visualization. At the browser provisions have been made to download the sequences. Highcharts and Highstock library were implemented for expression analysis chart, Scribl library for Annotation viewer and Linkurious based on SigmaJS library was used for network creation and visualization. Also, a user can perform sequence based search using two different search approaches: (i) Suffix tree based Bowtie search with mismatch provisions, and (ii) BLAST search.

Besides this, the portal provides facilities to explore the data according to gene ontology and clustering of the sRNAs. Gene Ontology information of each target gene has been provided. The results and data are displayed in the form of bubble chart containing the gene ontology slim IDs and the number of rsRNA:target interactions for the given ID. The size and color of the bubbles varies with respect to the number of rsRNA:target interactions. The selected ID redirects the user to a new page which contains the genes associated with the respective GO terms and targeting rsRNAs with provisions for exploration of expression analysis between a sRNA and target genes. Supplementary Figure S7 shows that from (A) GO bubble chart or table, (B) user gets redirected to respective page where the genes or rsRNAs involved could be selected. On the selection of a gene or rsRNA of interest a final plot opens (C) which shows expression of the target genes and targeting rsRNAs. From the list of genes and rsRNAs only the elements selected by the user are displayed. Besides these all, the portal also contains the details of various cluster analysis, from where again every individual sRNA could be picked and analyzed in depth.

In overall, this information portal provides a huge volume of experimental data in high quality visualization and representation to facilitate the user to have meaningful insights. All supporting materials of this study have also been hosted there. This portal will be also available at http://scbb.ihbt.res.in/SCBB_dept/Software.php.

## Supplementary Material

SUPPLEMENTARY DATA
