# Supplementary material for: A legion of potential regulatory sRNAs exists beyond the typical microRNAs microcosm
Source: Nucleic Acids Res. 2015 Oct 10;43(18):8713–24. doi: 10.1093/nar/gkv871 (PMC4605316; doi:10.1093/nar/gkv871)
Supplement: SUPPLEMENTARY DATA [file supp_43_18_8713__index.html]

A legion of potential regulatory sRNAs exists beyond the typical microRNAs microcosm — SUPPLEMENTARY DATA 

# A legion of potential regulatory sRNAs exists beyond the typical microRNAs microcosm

## SUPPLEMENTARY DATA

- SUPPLEMENTARY DATA
- SUPPLEMENTARY DATA
- SUPPLEMENTARY DATA
- SUPPLEMENTARY DATA
- SUPPLEMENTARY DATA
- SUPPLEMENTARY DATA
- SUPPLEMENTARY DATA
- SUPPLEMENTARY DATA
- SUPPLEMENTARY DATA
- SUPPLEMENTARY DATA
- SUPPLEMENTARY DATA
