# Supplementary material for: A legion of potential regulatory sRNAs exists beyond the typical microRNAs microcosm
Source: Nucleic Acids Res. 2015 Oct 10;43(18):8713–24. doi: 10.1093/nar/gkv871 (PMC4605316; doi:10.1093/nar/gkv871)
Supplement: SUPPLEMENTARY DATA [file supp_gkv871_nar-02020-n-2015-File007.doc]

**Supplementary materials**

**Supplementary Figure S1:** Distribution of known miRNAs in miRBase version 21 which display significant diversion from typical concept of canonical miRNA.

**Supplementary Figure S2:** Venn diagram representing target genes identified in AGO1-4 HITS-CLIP data and CLASH seq-data. Most of these rsRNA:target gene site (10,621) was identified in all the five methods

**Supplementary Figure S3:** Figure illustrate the target genes whose targeting rsRNAs have biogenesis locus on the respective opposite strand and the target binding sites and rsRNA biogenesis site coincides.

**Supplementary Figure S4:** Overall distribution and associations of rsRNAs regions. Large number of rsRNAs were found associated with complex repetitive elements.

**Supplementary Figure S5:** (A) Comparison of rsRNAs expression profile over the Alu consensus for normal and cancerous conditions across five individuals for BLCA cancer. The profile was found significantly different between the patient and normal samples. Alu elements host a number of such regulatory sRNAs. (B) Distribution of reads mapping on rsRNAs originating from repetitive elements normalized by totao number of repeat copy and average length of repeats.

**Supplementary Figure S6: Complete database structure of the information portal.**

**Supplementary Figure S7:** Figure describing the analysis performed in database. (A) GO bubble chart or table (any of the three GO category), (B) Expression of targeting rsRNAs and genes belonging to the selected GO category is displayed. (C) selection of rsRNA or gene gives full target genes/targting rsRNA expression details.

**Supplementary File 1:** Description and source of data for experiments used in this study.

**Supplementary File S2**: Expression details and sequences of primers used for calculating expression abundance of rsRNAs and target genes using qPCR. This file contains the expressions of rsRNAs and target genes in four cancerous cell lines. Each cell line have four replicates.

**Supplementary File S3:** Putative pre-miRNA candidates identified using DGCR8 CLIP-seq data. On these candidates the signature motifs of pri-miRNAs (31) were searched.

**Supplementary File 4:** List of common genes identified in AGO based HITS-CLIP data and CLASH data.

**Supplementary File 5:** List of rsRNAs found targeting genes and have significantly inverse correlation coefficient with protein expression.

**Supplementary File 6:** List of rsRNAs locus overlapping with Alus, Linc RNA, hAT-charlie and intronic locus.

**Supplementary File 7:** Distribution of mapped and unmapped sRNAs reported from various ncRNAs (36-39) on identified 11,234 rsRNAs.

**Supplementary File 8:** Top 10 significantly differentially expressed small regulatory RNAs in 19 cancer and respective normal states.

**Supplementary File 9:** List of most significantly enriched pathways affected by rsRNA-9881-n.
