# Supplementary material for: A legion of potential regulatory sRNAs exists beyond the typical microRNAs microcosm
Source: Nucleic Acids Res. 2015 Oct 10;43(18):8713–24. doi: 10.1093/nar/gkv871 (PMC4605316; doi:10.1093/nar/gkv871)
Supplement: SUPPLEMENTARY DATA [file supp_gkv871_nar-02020-n-2015-File008.pdf]

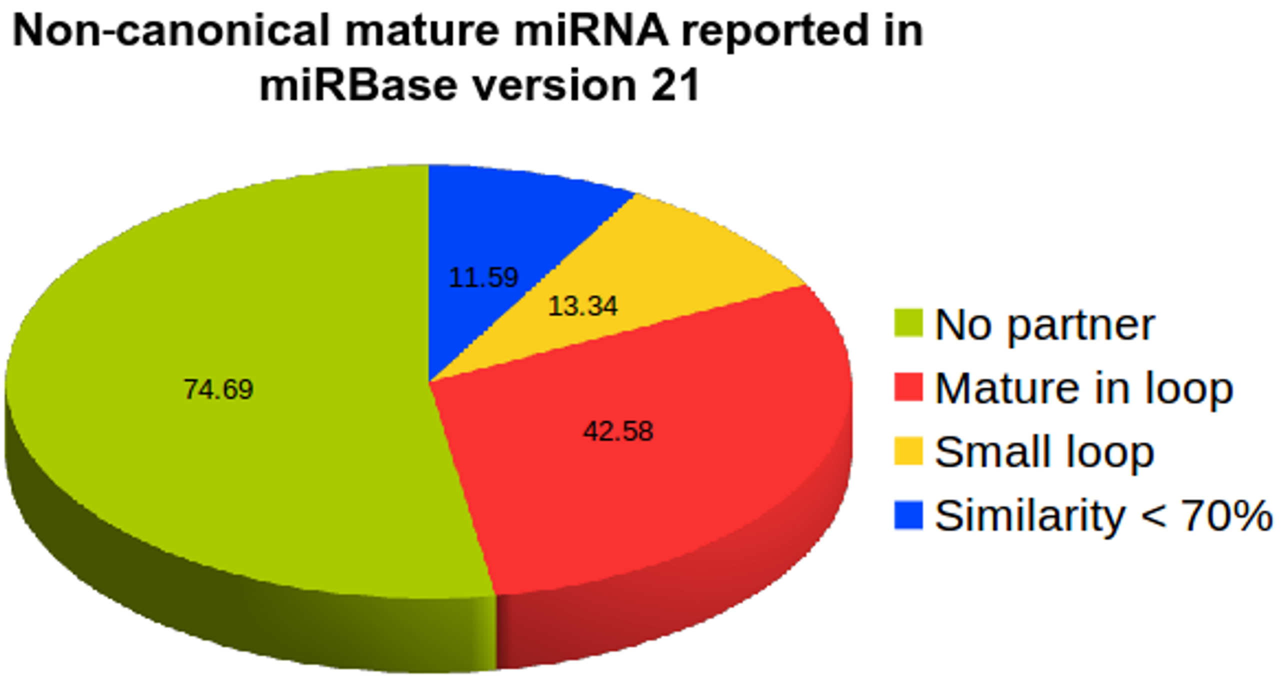

Supplementary Figure S1: Distribution of known miRNAs in miRBase version 21 which display significant diversion from typical concept of canonical miRNA.

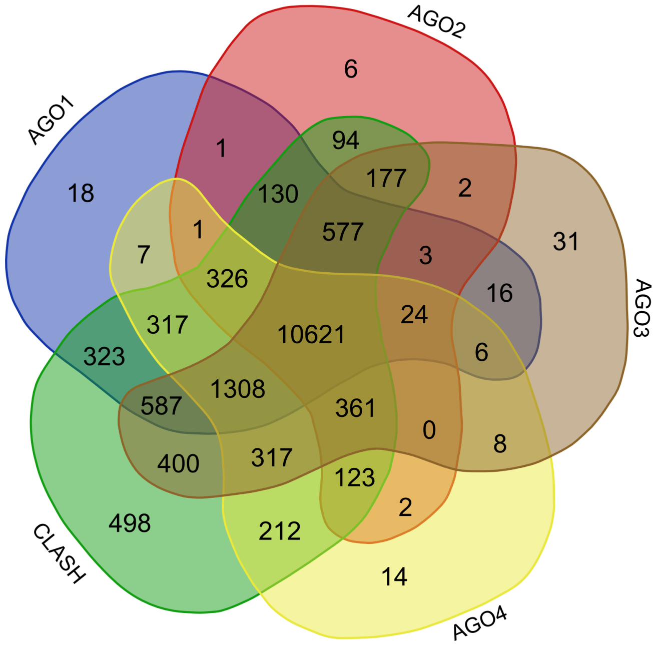

Supplementary Figure S2: Venn diagram representing target genes identified in AGO1-4 HITS-CLIP data and CLASH seq-data. Most of these rsRNA:target gene site (10,621) was identified in all the five methods

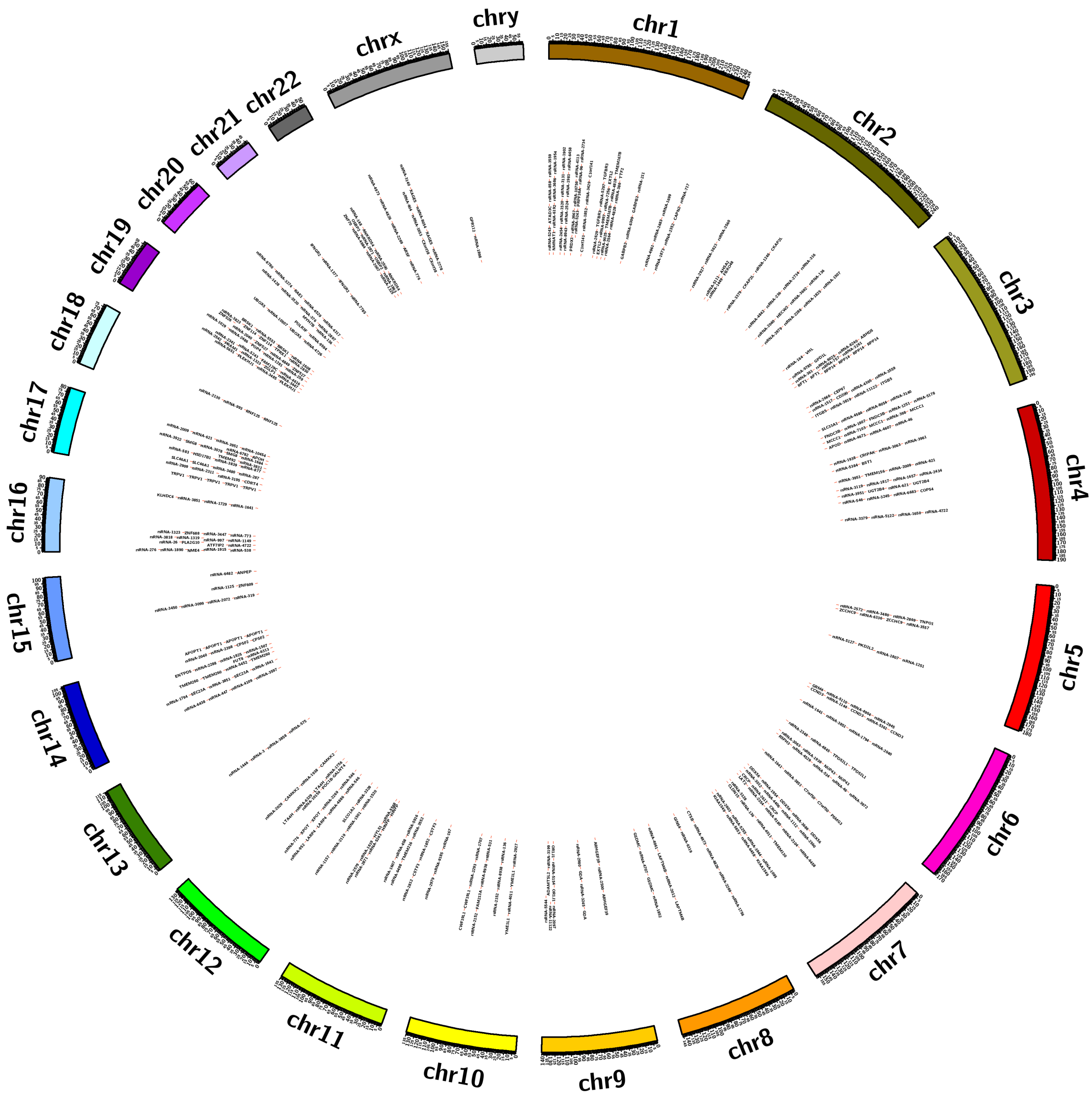

Supplementary Figure S3: Figure illustrates the target genes whose targeting rsRNAs have biogenesis locus on the respective opposite strand and the target binding sites and rsRNA biogenesis site coincide.

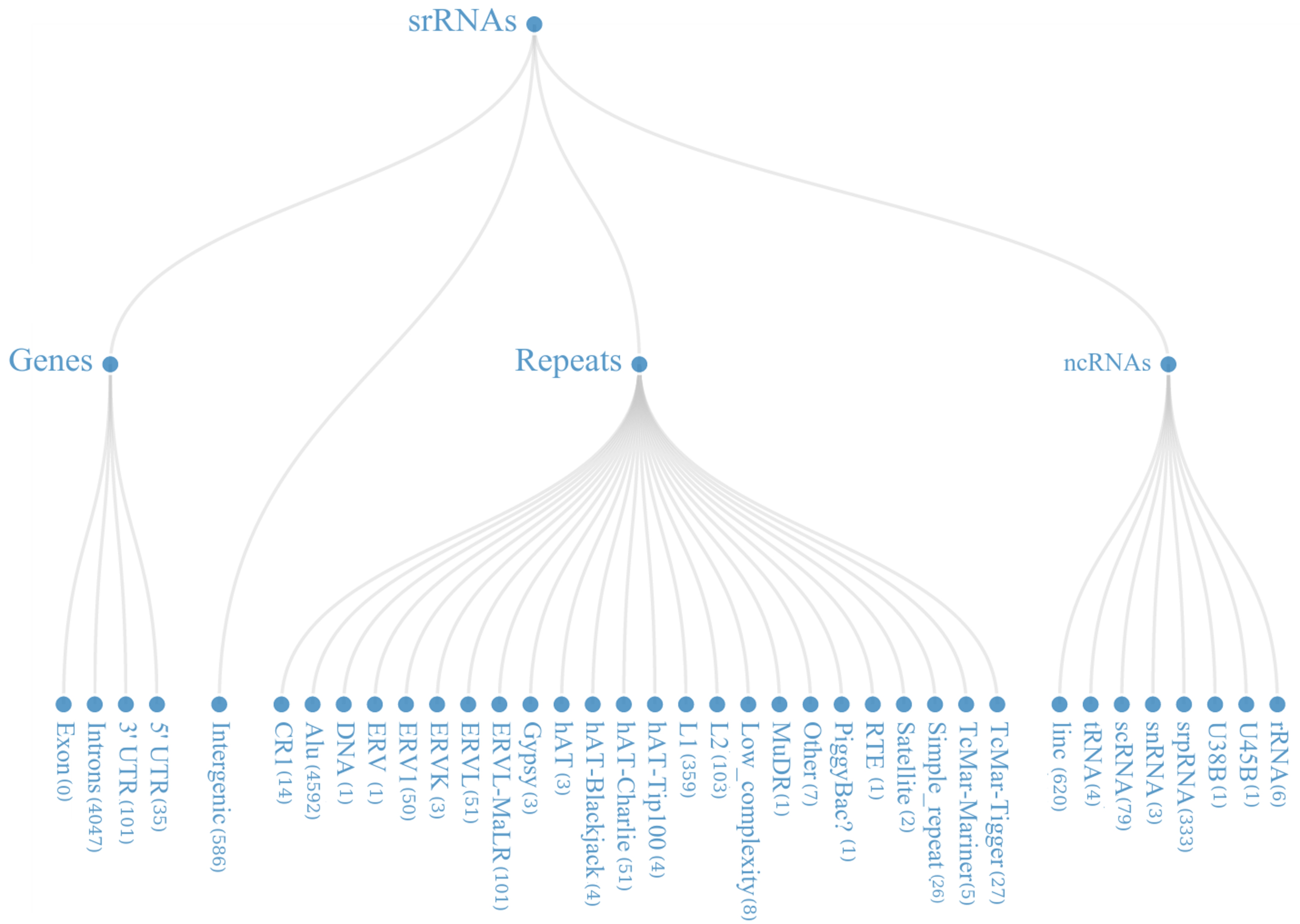

Supplementary Figure S4: Overall distribution and associations of rsRNAs regions. Large number of rsRNAs were found associated with complex repetitive elements.

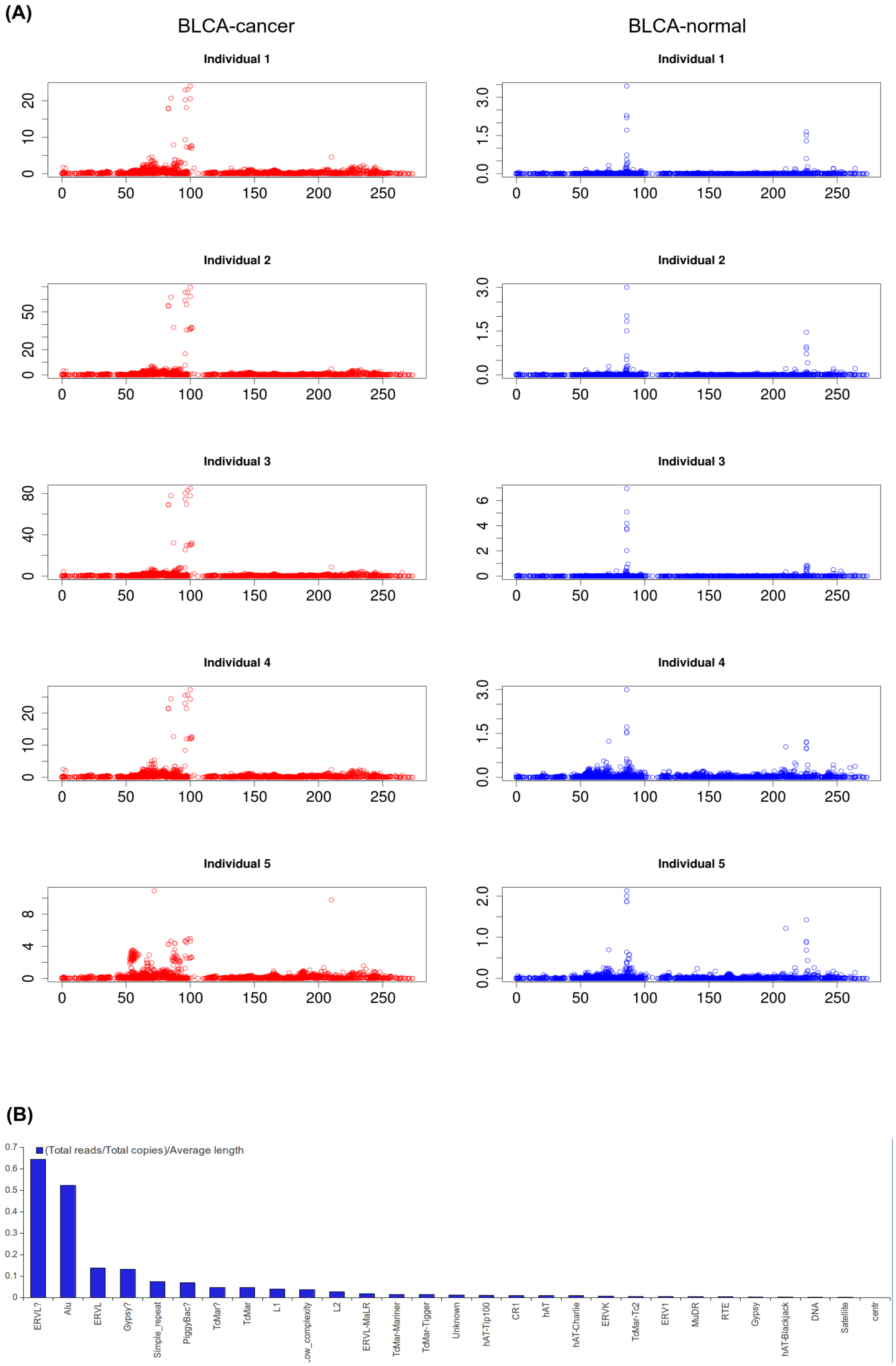

Supplementary Figure S5: (A) Comparison of rsRNAs expression profile over the Alu consensus for normal and cancerous conditions across five individuals for BLCA cancer. The profile was found significantly different between the patient and normal samples. Alu elements host a number of such regulatory sRNAs. (B) Distribution of reads mapping on rsRNAs originating from repetitive elements normalized by total number of repeat copy and average length of repeat.

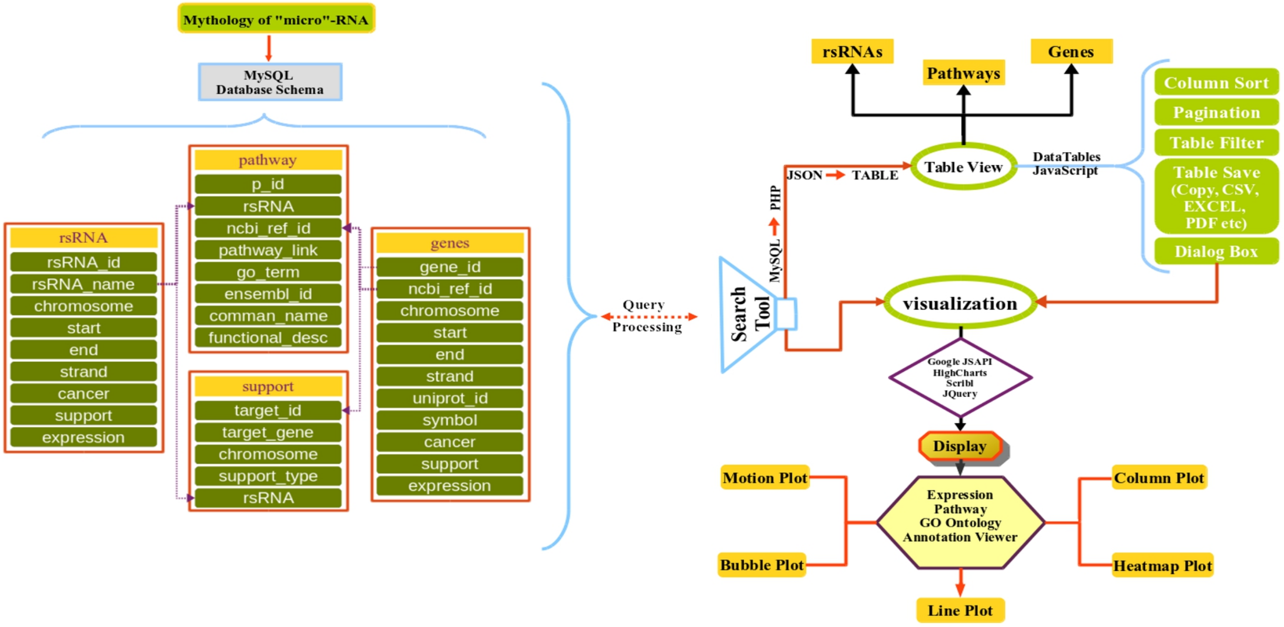

Supplementary Figure S6: Complete database structure of the associated information portal. The portal also provides a browser section, Bowtie suffix tree based and BLAST based quick search gateways. All the associated supplementary information have been made available at this portal page.

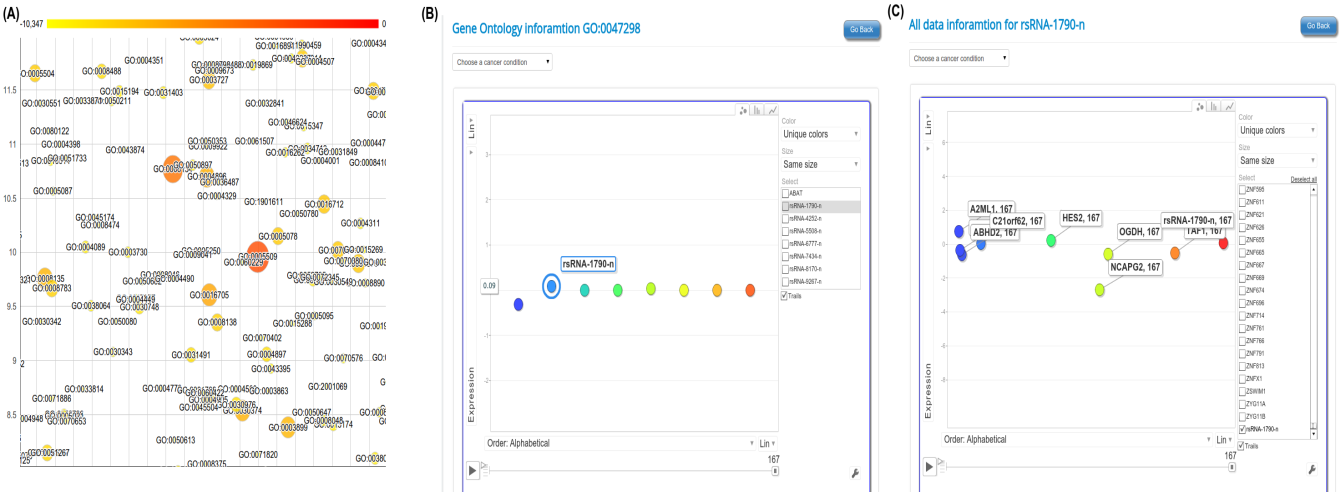

Supplementary Figure S7: Figure describing the analysis performed in database. (A) GO bubble chart or table (any of the three GO category), (B) Expression of targeting rsRNAs and genes belonging to the selected GO category is displayed. (C) selection of rsRNA or gene gives full target genes/targting rsRNA expression details.
