# Supplementary material for: A legion of potential regulatory sRNAs exists beyond the typical microRNAs microcosm
Source: Nucleic Acids Res. 2015 Oct 10;43(18):8713–24. doi: 10.1093/nar/gkv871 (PMC4605316; doi:10.1093/nar/gkv871)
Supplement: SUPPLEMENTARY DATA [file supp_gkv871_nar-02020-n-2015-File009.doc]

**Supplementary Table 1: Description and source of data for experiments used in this study.**

| **Cancer** | **Source** |
| --- | --- |
| Acute Myeloid Leukemia [LAML] | TCGA |
| Bladder Urothelial Carcinoma [BLCA] | TCGA |
| Brain Lower Grade Glioma [LGG] | TCGA |
| Breast invasive carcinoma [BRCA] | TCGA |
| Cervical squamous cell carcinoma and endocervical adenocarcinoma [CESC] | TCGA |
| Colon adenocarcinoma [COAD] | TCGA |
| Esophageal carcinoma [ESCA] | TCGA |
| Head and Neck squamous cell carcinoma [HNSC] | TCGA |
| Glioblastoma multiforme [GBM] | TCGA |
| Kidney renal clear cell carcinoma [KIRC] | TCGA |
| Kidney renal papillary cell carcinoma [KIRP] | TCGA |
| Lung adenocarcinoma [LUAD] | TCGA |
| Lung squamous cell carcinoma [LUSC] | TCGA |
| Ovarian serous cystadenocarcinoma [OV] | TCGA |
| Rectum adenocarcinoma [READ] | TCGA |
| Thyroid carcinoma [THCA] | TCGA |
| Uterine Carcinosarcoma [UCS] | TCGA |
| Kidney | SRP003902 |
| Adenoid_RNA | GSE29173 |
| Apocrine | GSE29173 |
| DCIS_RNA | GSE29173 |
| IDC | GSE29173 |
| Metaplastic | GSE29173 |
| Atypical_Medullary | GSE29173 |
| Adrenocortical carcinoma | SRP028291 |
